# Interest in Working as an Infection Prevention and Control Nurse and Perception of This Position by Nursing Students—Results of a Pilot Study

**DOI:** 10.3390/ijerph17217943

**Published:** 2020-10-29

**Authors:** Dorota Jaślan, Jerzy Rosiński, Małgorzata Siewierska, Anna Szczypta, Marta Wałaszek, Jadwiga Wójkowska-Mach, Agnieszka Gniadek, Renata Majewska, Anna Różańska

**Affiliations:** 1 Department of Infection Control and Mycology, Jagiellonian University Medical College, 31121 Krakow, Poland; dorota.jaslan@gmail.com (D.J.); mbmach@cyf-kr.edu.pl (J.W.-M.); 2 Faculty of Management and Social Communication, Institute of Economics, Finance and Management, Jagiellonian University, 30348 Kraków, Poland; jerzy.rosinski@uj.edu.pl; 3 Department of Ophthalmology, St. Rose Hospital, 30396 Krakow, Poland; tsiewi@wp.pl; 4 Faculty of Health and Medicine, Andrzej Frycz Modrzewski Krakow University, 30705 Kraków, Poland; infoepid@interia.pl; 5 Health Department, State Higher Vocational School, 33100 Tarnow, Poland; mz.walaszek@gmail.com; 6 Faculty of Health Science, Jagiellonian University Medical College, 31126 Kraków, Poland; agnieszka.gniadek@uj.edu.pl; 7 Department of Epidemiology, Jagiellonian University Medical College, 31034 Kraków, Poland; renata.majewska@uj.edu.pl

**Keywords:** infection control, students, nursing, infection control practitioners, patient safety, attitude of healthcare personnel

## Abstract

**Background:** The results of several studies in the area of infection control in Poland are disturbing. The situation may be shaped by many factors. However, the key factor for effective infection prevention and control is dedicated personnel, especially infection prevention and control nurses (IPCN). Nevertheless, based on the available published data and the authors’ experience, in many Polish hospitals infection control is not sufficiently appreciated by managers, it is consequently underfunded, and treated by medical staff as a nuisance. This may influence the nurses willingness to work as IPCN. The aim of the study was to assess the nursing students’ perception of the work of IPCN and their interest in employment in this position, as well as the potential reasons for choosing this particular specialization. **Materials and methods:** The study was conducted using the authors’ anonymous questionnaire conducted among nursing students of three Polish universities. The questionnaire was prepared by a panel of experts working in the field of infection control, including nurses working both as academic teachers and infection control nurses in hospitals. The design of the questionnaire was based on the authors’ own experience, knowledge, and exchanging information with the practitioners in infection control in Poland. The reliability of the questionnaire was confirmed by the Cronbach alpha test. The raw alpha values and 95% CI for two main questions concerning opinion were: 0.76 (0.72–0.81) and 0.69 (0.63–0.75). **Results:** The study was conducted among 253 students, mostly women (98%) of full-time (31.4%) and extramural (68.6%) studies. The age range of the respondents was 20–58 years, median = 26 years, IQR = 19 years. To the key item in the questionnaire, i.e., “Would you like to work as an IPCN?”, 84.6% (214 respondents; first group) of the respondents answered “no” and 15.4% (39 respondents, second group) answered “yes”. The results revealed no significant differences between the two groups concerning the position responsibilities and appreciation by other medical staff. Additionally, for respondents willing to work as ICPN the most important issues were the influence on patient safety, expected salary, and possibility of professional development; for the respondents from the other group the most important issue was lack of contact with patients. The results concerning the students’ opinion on the perception of IPCN by medical personnel proved to be peculiar. About 80% of the respondents confirmed the IPCNs’ key role in ensuring patient and personnel safety, while only 31.6% declared their high standing in the hospital hierarchy. **Conclusions:** The obtained results indicate the necessity of thorough studies on the organization and structure of infection control in Polish hospitals, with a particular emphasis on building a positive perception of IPCNs by medical staff, as well as implementing an education campaign on infection control in the hospital environment.

## 1. Introduction

Infection control nursing is one of the specializations that nursing graduates can choose in Poland. It is different from other specialties due to the scope of duties and its position in the organizational structure. In modern healthcare, the history of this nursing specialty dates back to 1960’, to the beginnings of systemic infection control and prevention in the United States [1]. The beginnings of modern surveillance of hospital infections in Poland date back to the turn of the 21st century [2]. In the era of increasing number of healthcare-associated infections and antimicrobial resistance, infection prevention and control play a key role in hospitals and other healthcare facilities. Effective infection prevention and control programs ensure the safety of patients and healthcare workers in endemic situations, with individual, limited outbreaks, but are particularly important during an epidemic or a pandemic. Effective surveillance of infections is based primarily on competent staff: infection control and prevention nurses (ICPN), physicians and microbiologists, comprising infection control teams (ICT). In ICT the key role is taken on by ICPNs. Their responsibilities include many tasks that require collaboration with various professional groups within the hospital [3,4]. However, infection control and prevention nurses claim that they frequently experience problems in cooperation with various groups of hospital workers and that the changes that are taking place with respect to infection control are not positive [5,6]. The confirmation that cooperation with the ward personnel is particularly challenging results from the studies in the area of health-care associated infections epidemiology, suggesting that the environment in Polish hospitals is not friendly to infection control. For example, despite two decades of implementation of up-to-date surveillance of hospital-acquired infections, some of the highest incidence rates are recorded with respect to *Clostridium difficile* and the proportion of multi-drug resistant strains isolated from invasive infections [7,8]. There is also an alarming epidemiological situation with regard to bloodstream infections and, probably, ineffective registration of infections [9,10]. The effective implementation of strategy and procedures in the area of infection control and their surveillance in Polish hospitals, where the culture of the organization is characterized by a high power distance, masculinity, and a high uncertainty avoidance index, is a particularly difficult task [11].

Working as an IC nurse requires knowledge of infection control and prevention, including epidemiology and microbiology, which are not explored thoroughly enough in the vocational education program. It is not until the specialized education that the nurses’ qualifications in this field improve, which is why the completion of such education is required by law from nurses employed as infection control nurses [12]. The post-graduate specialization in this case lasts two years. Working as an ICPN requires not only specialized knowledge of epidemiology and microbiology, but also statistics, psychology, and above-average interpersonal skills. Such broad background is needed because among the ICPN responsibilities there are: infection detection, registration, analyzing and reporting, developing and implementation of preventive procedures, staff education, internal audit in the area of infection control, etc. Billing et al. indicated six future-oriented competency domains for an infection preventionist (also for IPCN): Leadership, professional stewardship, quality improvement, IPC operations, IPC informatics, and research [13]. These challenges are of a different kind than for other nursing specialists and taking into account conditions and circumstances of infection control and prevention in Poland, the assessment of perception of NS of ICPN and the willingness to work in this position is fully justified.

Polish nurses working as infection control nurses declared in the questionnaire that in everyday practice they devote approximately one third of their work time to monitoring infections, approximately 10% of their work time to an internal audit of procedures related to infection control, and another 10% to the development and implementation of procedures, as well as staff training. ICPNs estimated their influence over the implementation of individual tasks and independence of decision-making only at about 65–70%, depending on the tasks and types of activities, as well as the size of the hospital. In addition, almost half of the respondents declared that during their professional work as an epidemiological nurse, adverse changes have occurred [5]. At the same time, most of the respondents declared significant difficulties in cooperation with the medical personnel, administrative staff and the microbiological laboratory [6]. These difficulties were not described precisely in this questionnaire, but the survey was only a preliminary measure to assess the situation. However, based on the results of the cited study (not fully published) and the authors’ experience, in many (or even most) Polish hospitals infection control is not sufficiently appreciated by managers. It is consequently underfunded, and treated by medical staff as a nuisance. This may influence the nurses willingness to choose this position and specialization. 

The objective of this study was to assess the nursing students’ perception of the work as ICPN and their interest in employment in this position, as well as the potential reasons for choosing this particular specialization.

## 2. Material and Methods

The study was conducted between October and November 2016 using an anonymous questionnaire among nursing students of three universities in southern Poland, i.e., students of the Krakow Academy of Andrzej Frycz Modrzewski (Faculty of Health Care), students of the State Higher Vocational School in Tarnów (Institute of Health Care), and students of the Faculty of Health Sciences of the Jagiellonian University Collegium Medicum. The Krakow Academy of Andrzej Frycz Modrzewski is a private university with a total number of 500 nursing students, the State Higher Vocational School in Tarnów and Jagiellonian University are public schools, with the total numbers of 220 and 630 nursing students, respectively. Jagiellonian University is the oldest university in Poland founded over 600 years ago while the other schools were founded in the late 1990s. The questionnaire was prepared by a panel of experts working in the field of infection control, during a two-stage process. Firstly, three scientists with experience and interest in the area of infection control, including a registered nurse, two nurses working in infection control, and a prevention nurse with additional academic activity proposed the tool frame and answers. Justifications (answers) for interest (or lack of it) in working as an infection prevention and control nurse have been chosen based on the authors’ experience gained through participation in a scientific project in the field of infection control and exchanging information with practitioners in Poland. Then, two people with academic experience working as ICPNs reviewed the questionnaire, proposed some modifications from the practitioner’s perspective, and by way of discussion a final version of the tool was drawn up. The questionnaires (paper forms to fill in) were personally distributed among students by authors after classes, which was possible thanks to the great appeal of the classes among study groups having courses with authors during the study period. Thus, the choice of the study group was selective. It resulted in a random selection that concerned only students of specific groups in each university, but without stratified-cluster sampling. The questionnaire was distributed together with the information about the aim of the study. It mentioned that participation is voluntary and anonymous, as required by the Bioethical Committee of Jagiellonian University for such studies. The study comprised about 21.8% nursing students from Krakow Academy, 11.4% from Higher Vocational School in Tarnów, and 12.2% from Jagiellonian University Medical College.

The questionnaire consists of seventeen questions: Twelve concerned the demographic characteristic of respondents, i.e the independent variables such as age, gender, total length of nursing internships completed within the course of studies, type of studies (bachelor’s, master’s, or the “bridging programme”), specialization, work experience.

The following questions aim to find out whether the respondents are interested in working as an infection control nurse, what factors does their decision rely on, and what is their opinion on the role of IPCN and on the perception of their work by the medical personnel. Respondents who gave a positive answer to the question whether they were willing to work as an infection control nurse were asked to name their main reasons for such a decision—13 different justifications were proposed in the questionnaire. Respondents who did not want to work as a nurse epidemiologist also had the opportunity to justify their answer—the questionnaire contained 10 statements proposed by the authors of the study. Individual justifications in favor of or against working as an infection control nurse are detailed in the description of the results. Each of the listed categories was to be assessed on a Likert scale from “very significant impact” (5 points) to “definitely no impact” (1 point). There were no open questions in the questionnaire. The questionnaire was evaluated in terms of compliance with the main rules for questionnaire construction, i.e.,: simple vocabulary, avoiding words that are abstract, not fully defined or ambiguous, jargon, moralizing language, avoiding negations, names of institutions, surnames, too long items, and covering more than one issue. The evaluation was carried out by an expert from the Department of Management and Social Communication of Jagiellonian University. [14]. Additionally, Cronbach’s alpha test was conducted. The reliability proved to be satisfactory, for questions no. 13 and 14: the raw alpha values and 95% CI were: 0.76 (0.72–0.81) and 0.69 (0.63–0.75), respectively. Seven common categories of the respondents’ justification were selected: salary, possibility of professional development, possibility of promotion, contact with patients, responsibilities of the position, impact on patient safety, and appreciation by other medical staff were chosen to be compared between the two groups of students willing to work as ICPN and those not willing to.

The statistical analysis shows descriptive statistics of respondents’ characteristics and subgroup comparisons. The descriptive analysis of qualitative characteristics was carried out by calculating the number and percentage of occurrences for each value. To characterize the age and seniority of the respondents, its quartiles distribution were presented due to a skew distribution. The analysis of differences between the analyzed subgroups was carried out with the Pearson chi-square or exact Fisher test (if more than 20% of cells have expected values lower than 5) for qualitative characteristics and the Mann-Whitney test for quantitative ones. Univariate and multivariate logistic regression models were used to identify factors associated with the willingness to work as an ICPN. The level of significance was assumed as *p* < 0.05.

The analysis employed the following statistical software: IBM SPSS (Statistical Package for the Social Sciences—SPSS) STATISTICS 24, Armonk, NY, USA and Microsoft Excel Microsoft Office 2016 Redmond, WA, USA.

The study was approved by the Bioethics Committee of the Jagiellonian University, decision number 122.6120.124.2016.

## 3. Results

The study was conducted among 253 students (5 men and 248 women), most of them were extramural students: 68.8% vs. full-time: 31.2%. The age range of the respondents was 20–58 years, median = 26 years, IQR = 19 years. Most of them were Master’s degree students (81.8%) who worked as nurses (82.3%) with work experience ranging from 1 month to 37 years (median: 10 years). To the key item in the questionnaire, i.e., “Would you like to work as an epidemiological nurse?”, 84.6% (214 respondents) of the respondents answered “no” and 15.4% (39 respondents) answered “yes”. Only seniority in the profession of a nurse was a factor that significantly correlated with the declaration of willingness to work as an infection control nurse. While taking into account the positive and negative responses to the question of willingness to work as an infection control nurse, the demographic characteristics of the respondents are presented in Table 1 and Table 2. Data presented in Table 1 and Table 2 do not include missing answers for each category.

The vast majority of respondents admitted that, in their opinion, the work of an ICPN is of key importance in ensuring patient safety and that it is helpful in providing safe work conditions for the staff of the ward, and that ICPNs are helpful in implementing procedures, respectively—79.4%, 83.4%, and 84.6% of the respondents (Table 3). At the same time, 52.6% of the respondents declared that the work of an ICPN is burdensome for the personnel of the wards and only 31.6% concluded that work as an ICPN enjoys great respect among the hospital staff. Such opinions were significantly more often expressed by respondents with a shorter work experience. The opinion that work as an ICPN enjoys great respect among the hospital staff was also more often expressed by the respondents from the Krakow Academy—the school with a greater proportion of students participating in the study. There was no significant correlation between the willingness to take up work as an ICPN and the distribution of answers to most questions, except the one about respect for the ICPN position. A significant correlation in some subcategories concerned the questions declaring the role of ICPN for the safety of patients and staff, as well as the ones on their respect among the personnel and burdensomeness for the ward. No statistical differences were found in the case of an opinion on ICPN being helpful in ensuring safe work for the staff of the ward (Table 3).

Factors significantly correlating with the type of response to the majority (3 or 4 out of five) of questions about the perception of the work of ICPNs was also the respondents’ age and work experience. Overall, people aged 40 years and older and with work experience of more than 10 years perceived the work of ICPNs more favorably. They declared more frequently that their work enjoys great respect among hospital staff, is of key importance for patient safety, and is helpful in implementing procedures.

The respondents declaring willingness to work as IPCN indicate following items as the most important reasons for such a decision (“a significant impact”): The opportunity to cooperate and interact with various professional groups in the hospital, influence on improving patient safety, the fact that the position is located high in the hospital hierarchy, the opportunity for promotion (Table A1). The respondents who were not interested in working as IPCN justified their answers by the lack of/limited contact with patients and the fact that this work is associated with a greater burden of administrative procedures (Table A2). The analysis—focused on seven categories of justifications for willingness (or its lack) to work as an ICPN—revealed no significant differences between the two groups in terms of position responsibilities and appreciation by other medical staff (Table 4). Additionally, for respondents willing to work as ICPN the most important issues were influence on patient safety, expected salary, and possibility of professional development; for the respondents from the other group the most important issue was lack of contact with patients (Table 5).

## 4. Discussion

There were 1011 nurses, i.e., 0.4% of all nurses and 1% of practicing nurses, working in infection control in Poland in 2014 [15]. Results from a point prevalence survey (PPS) that was conducted in Poland in the framework of an ECDC (European Centre for Disease Prevention and Control) programme in 2012 show that there was 1 IPCN per 250 beds on average [16] and a similar rate was confirmed in the questionnaire study PROHIBIT [17]. The current Polish legal regulations require the employment of 1 IPCN per 200 beds [4]. According to the data from Statistics Poland in 2017, there were 957 hospitals in Poland with 187,000 beds [18], which means that there is an average of one IPCN per about 180 beds. One could therefore conclude that a shortage of IPCN who meet the criteria for working in this position in Polish hospitals is probably insignificant. A separate issue, however, is whether the nurses that have such a specialization want to work as infection prevention and control nurses. In the years 2002–2018, 2078 nurses and midwives obtained the title of a specialist in infection prevention and control (epidemiological nurse) [19], which in view of the data quoted above, indicates that about half the nurses with a specialist title are in fact employed in this position. A similar phenomenon is observed in the case of employment of nursing graduates in general—only 60% of them do it [20]. Considered together with data on the average age of IPCNs in Poland amounting to 49 years, the need for a constant inflow of new candidates for the profession becomes apparent [21]. Only 15% of the respondents declared their willingness to work as IPCN, which points to the fact that such a position is unappealing to nurses [21]. Nurses that are new to the profession are interested in gaining professional experience through working with patients. Such experience constitutes a bargaining chip when simultaneously applying for jobs in many healthcare institutions. Additionally, unfortunately, seniority was a factor that correlated with the desire to take up work as an IPCN. The average work experience among the respondents that declared their willingness to work in the profession was much longer, i.e., 11 years longer, than the one among the people who were uninterested in it. Perhaps the direct reason behind it is the nature of the work as an IPCN, which is not associated with intense contact with patients. Hence, it could be an alternative in the case of burnout or fatigue from work in the position of a staff nurse [21]. Załuski and Makara-Studzińska, in their study of the relationship between burnout, emotional work, and commitment to work among health care workers found 33% of nurses to exhibit a high level of negative changes in their relations with the patient and 17% to display a high level of psychophysical exhaustion [22]. Withdrawal of a worker from a relationship with the patient is also considered by Maslach to be a defence mechanism in the event of depletion of psychophysical resources [23]. Deterioration of relationships with patients, but also with colleagues and managers was indicated by Irinyi et al. as an important issue in nurses’ burnout [24]. Nursing burnout is related to factors in the work environment, but the situation is more complex: factors that influence burnout depend on the stage of burnout. Firstly, socio-demographic factors, such as: type of service, work experience, civil status, working shift, and the type of contract play a key role in the process. Secondly, depersonalization and personal accomplishment are also factors that deserve due consideration [25]. Changing the scope of duties or the nature of work is a frequently used remedial tool in the case of burnout syndrome at the personal accomplishment stage, which may explain the interest in working as an ICPN declared by respondents with a greater job experience in our study.

According to the students, working as an IPCN is associated with, on the one hand, lack of contact with the patient and, on the other, working in an interdisciplinary team which gives one a possibility to cooperate and stay in contact with various professional groups in the hospital, and can therefore constitute an appealing alternative to direct work with the patient in the case of emotional and psychophysical fatigue. It is interesting that positive answers concerned the improvement of the patient’s safety (almost 90% of the responses), but at the same time, almost half of the respondents who were reluctant to work as an IPCN stated the opposite, i.e., that an ICPN has little impact on improving this safety. It is difficult to assess the reasons behind this opinion of the participants of this study, i.e., the insignificant effect on improving patient safety. Such a result is nevertheless consistent with the limited decision-making influence that nurse epidemiologists enjoy in Polish hospitals. IPCNs in Poland have a very limited decision-making authority, so they determine the epidemiological safety of the patient to a very small degree. It is clear from the study conducted in 2014 that IPCNs themselves assess their decision-making to be at the level of 60% [5]. In the generally accepted management theory, the effectiveness of the tasks performed in most professions is conditioned by the fact that the responsibilities imposed on/delegated to a given profession should go together with empowerment of the people who perform the given tasks. Empowerment (which involves decision-making) has simultaneously an impact on respect in the work environment. Therefore, the fact that over 50% of our respondents mentioned these reasons (this work is not appreciated by other medical workers: 53.9%, it has little influence on the real/day-to-day work in the wards: 53.3%) as the cause for their reluctance to take up work in the profession of an IPCN is directly associated with insufficient empowerment of this profession in regards to the autonomy and greater independence in decision-making.

One of the most important and disturbing results of this study is the fact that only around 30% of respondents declared that the work of an ICPN enjoys great respect among hospital staff, despite the fact that the majority of them consider this work to influence the improvement in the patient and staff safety. The study was conducted on a relatively small group of nursing students, the majority of whom, however, were working as nurses and were, therefore, familiar with the realities of working in Polish hospitals. Hence, a hypothesis can be put forward that these results are a good reflection on the atmosphere and organizational conditions which the Polish infection control teams, especially IPCN, operate in. The results of research on the epidemiology of hospital-acquired infections in Poland, as well as on the knowledge and practice of hand hygiene among nurses and physicians, confirm that infection control, including the work of epidemiological nurses, is not appreciated enough. This is probably, among other things, due to the organizational culture of Polish hospitals, which are typically highly hierarchical organizations, with a great deal of power distance, masculinity, and a high uncertainty avoidance index, i.e., places in which nurses who are predominantly women do not enjoy high standing, and, consequently, their influence on effective implementation of infection control programs is limited [7]. The lack of recognition for the position of an IPCN by other medical workers may be also related to the flawed organization of the human resource management system in Polish hospitals and the reluctance to condemn leaders among health care workers from the nursing staff. Changing the system of undergraduate education of nurses and midwives, with a particular emphasis on teaching subjects, shaping a positive image of the IPCN as a leader, may improve the opinion of medical workers of this position, and, consequently, enhance the working standard for IPCNs. The appearance of subjects such as human capital management, coordinated care or, finally, epidemiological nursing in the curricula of medical education could positively influence the image of an epidemiological nurse in the health care system. The post-graduate education of nurses and midwives is also important, but in the case of clinical practice, it is a challenge to the whole health care system in Poland in regard to the necessary changes in organizational culture.

On the other hand, a positive observation is the fact that older respondents and those with longer seniority declared a little more frequently that ICPNs enjoy great respect among the staff and less often stated that their work is burdensome for the wards. Perhaps, in their professional career, they more often encountered situations in which IPCNs turned out to be helpful.

It is worthwhile to emphasize the fact that the knowledge of the work of an IPCN is not widespread, which is evidenced by the high percentage of the answers “it is difficult to say” in the survey. They amounted to as many as 30–40% of the answers among the participants who had a positive attitude and 20–30% of others.

Obviously, it cannot be categorically said that giving the answer of “it is difficult to say” is associated with little knowledge of the work of an IPCN. The respondents, in the course of their professional activities, might have worked mainly in ambulatory care or in spite of the knowledge that they have on IPCN specialization they could not determine the extent to which a given reason influences their decision. However, regardless of the reasons for this, there should be an active dissemination of knowledge about the importance of the profession of an IPCN (clear expectations associated with it, transparent empowerment in the organization). Action taken in this field will have influence on greater awareness regarding the decisions made by students.

## 5. Limitations of the Study

Due to the way of sampling that did not involve a stratified-cluster design, there is probably a selection bias connected with a slight over-representation of participants from one university. Additionally, the process of validation of the questionnaire did not involve testing on the small group of potential respondents, and as a result this should be taken as a pilot study. Moreover, we performed this study four years ago, in an environment free from pandemic circumstances. Today, the results could be completely different, because the infection control and prevention team’s role in assuring patient and personnel safety is probably appreciated much more than a few years ago.

## 6. Conclusions

Only about fifteen percent of respondents declared interest in working as an infection prevention and control nurse. For those who were interested in the opportunity to cooperate and interact with various professional groups in the hospital, influence on improving patient safety and the fact that the position is located high in the organizational hierarchy of hospitals were the most important justifications. However, both in the case of respondents potentially interested and not interested in working as an IPCN, about one third was not able to decide on specific justifications. In the opinion of the majority of respondents, the work of IPCNs has an impact on improving the safety of patients and staff, but about half stated that it is burdensome for the functioning of wards and only one third concluded that the work of ICPNs enjoys great respect among the hospital staff. The problem raised in this study requires more thorough research involving a larger population and additionally the implementation of qualitative methods (e.g., in-depth individual interview) and these results should be considered preliminary to further research.

## Figures and Tables

**Table 1:** Respondents’ declaration of willingness to work as an infection control nurse depending on sex, type and mode of study, and professional experience.

Respondents Characteristics		Would You Like to Work as an Epidemiological Nurse?				*p*
		no		yes		
		*n*	%	*n*	%	
Gender	female	209	85.0	37	15.0	*n*/s
	male	4	80.0	1	20.0	
	total	213	84.7	38	15.3	
Mode of studies (full-time or extramural)	full time	71	91.0	7	9.0	*n*/s
	extramural	141	82.0	31	18.0	
	total	212	84.8	38	15.2	
Degree (bachelor’s, master’s, or “bridging programme”)	bachelor’s	16	100.0	0	0.0	*n*/s
	master’s	162	83.9	31	16.1	
	bridging programme	23	85.2	4	14.8	
	total	201	85.2	35	14.8	
University	Krakow Academy	109	83.6	21	16.4	*n*/s
	State Higher Vocational School in Tarnów	25	92.6	2	7.4	
	Faculty of Health Sciences of the Jagiellonian University Collegium Medium	77	85.6	13	14.4	
	total	211	85.4	36	14.6	
Has already worked as a nurse?	no	39	90.7	4	9.3	*n*/s
	yes	166	83.0	34	17.0	
	total	205	84.4	38	15.6	
Specialization yes/no	no	130	85.5	22	14.5	*n*/s
	yes	34	77.3	10	22.7	
	total	164	83.7	32	16.3	

*N*: Numbers. *P*: Level of significance; *n*/s: Not significant. Numbers in rows marked as “total” do not sum up to 253 due to missing answers in some questions.

**Table 2:** Respondents’ declaration of willingness to work as an infection control nurse depending on age, practical training, and work experience.

Respondents Age and Professional Experience	Would You Like to Work as Infection Prevention and Control Nurse?								*p*
	no				yes				
	Median	Q1	Q3	*n*	Median	Q1	Q3	*n*	
Age (years)	25	23	41	210	33	24	45	38	*n*/s
total length of nursing internships completed during studies	2200	250	2500	137	960	160	2200	25	*n*/s
work experience [years]	8.00	1.00	21.00	166	19.00	0.83	25.00	34	0.049

*p*: Level of significance from the Mann-Whitney test; *n*/s: Not significant.

**Table 3:** Respondents’ opinion on the role of infection prevention and control nurse depending on type of studies, age, work experience, and declaration of the interest in work at this position.

Respondents’ Characteristics	The Work of an ICPN is of Key Importance in Providing Patient Safety				ICPNs are Helpful in Implementing Procedures				The Work of an ICPN is Helpful in Ensuring Safe Work Conditions for the Staff of the Ward				The work of ICPNs Enjoys Great Respect among Hospital Staff				The Work of ICPNs is Burdensome for the Staff of the Wards			
	no answer	no	yes		no answer	no	yes		no answer	no	yes		no answer	no	yes		no answer	no	yes	
Type of studies	*n*/%	*n*/%	*n*/%	*p*	*n*/%	*n*/%	*n*/%	*p*	*n*/%	*n*/%	*n*/%	*p*	*n*/%	*n*/%	*n*/%	*p*	*n*/%	*n*/%	*n*/%	*p*
Bachelor’s	4/20.0	1/5.0	15/75.0	0.048	3/15.0	2/10.0	15/75.0	*n*/s	3/15.0	2/10.0	15/75.0	*n*/s	3/15.0	10/50.0	7/35.0	*n*/s	4/20.0	8/40.0	8/40.0	*n*/s
Master’s	9/4.4	34/16.6	162/79.0		12/5.9	21/10.2	172/83.9		11/5.4	24/11.7	170/82.9		12/5.9	133/64.9	60/29.3		12/5.9	82/40.0	111/54.1	
Bridging programme	2/7.1	2/7.1	24/85.7		1/3.6	0/0.0	27/96.4		1/3.6	1/3.6	26/92.9		1/3.6	14/50.0	13/46.4		1/3.6	13/46.4	14/50.0	
Universities																				
Krakow Academy	12/8.9	6/4.4	117/86.7	<0.001	13/9.6	6/4.4	116/85.6	0.011	11/8.1	10/7.4	114/84.4	*n*/s	12/8.9	65/48.1	58/43.0	<0.001	14/10.4	63/46.7	58/43.0	0.008
Higher Vocational School in Tarnów	0/0	3/11.1	24/88.9		0/0.0	3/11.1	24/88.9		0/0	2/7.4	25/92.6		0/0	21/77.8	6/22.2		0/0	9/33.3	18/66.7	
Jagiellonian University Medical College	3/3.3	28/30.8	60/65.9		3/3.3	14/15.4	74/81.3		4/4.4	15/16.5	72/79.1		4/4.4	71/78.0	16/17.6		3/3.3	31/34.1	57/62.6	
Age																				
Less than 40 years	6/4.3	29/21.0	103/74.6	0.003	5/3.6	17/12.3	116/84.1	0.018	6/4.3	19/13.8	113/81.9	*n*/s	6/4.3	99/71.7	33/23.9	0.002	6/4.3	52/37.7	80/58.0	*n*/s
40 years or more	9/8.0	7/6.3	96/85.7		11/9.8	5/4.5	96/85.7		9/8.0	8/7.1	95/84.8		10/8.9	56/50.0	46/41.1		11/9.8	51/45.5	50/44.6	
Work experience in years																				
No data	5/9.1	7/12.7	43/78.2	0.001	4/7.3	4/7.3	47/85.5	0.005	4/7.3	6/10.9	45/81.8	*n*/s	4/7.3	40/72.7	11/20.0	0.006	4/7.3	17/30.9	34/61.8	0.030
Less than 10 years	1/1.0	24/24.2	74/74.7		1/1.0	15/15.2	83/84		2/2.0	15/15.2	82/82.8		2/2.0	68/68.7	29/29.3		2/2.0	41/41.4	56/56.6	
10 years and more	9/9.1	6/6.1	84/84.8		11/11.1	4/4.0	84/84.8		9/9.1	6/6.1	84/84.8		10/10.1	49/49.5	40/49.5		11/11.1	45/45.5	43/43.4	
Declaration of the interest in work as ICPN																				
no	13/6.1	36/16.8	165/77.1	*n*/s	12/5.6	20/9.3	182/85.0	*n*/s	12/5.6	25/11.7	177/82.7	*n*/s	13/6.1	146/68.2	55/25.7	<0.001	13/6.1	83/38.8	118/55.1	*n*/s
yes	2/5.1	1/2.6	36/92.3		4/10.3	3/7.7	32/82.1		3/7.7.	2/5.1	34/87.2		3/7.7	11/28.2	25/64.1		4/10.3	20/51.3	15/38.5	
total	15/5.9	37/14.6	201/79.4		16/6.3	23/9.1	214/84.6		15/5.9	27/10.7	211/83.4		16/6.3	157/62.1	80/31.6		17/6.7	103/40.7	133/52.6	

*N*: Number; *n*/*s*: Not significant.

**Table 4:** Impact of the considered issues on the willingness to work as an infection control and prevention nurse.

Reason for the Justification of Willingness to Work as an ICPN	Would You Like to Work as an Infection Control and Prevention Nurse?			*p* Value
	Total *n* (%)	Yes *n* (%)	No *n* (%)	
the amount of remuneration	61 (24.8%)	19 (51.4%)	42 (20.1%)	<0.001
possibility of professional development	144 (58.1%)	31 (81.6%)	113 (53.8%)	0.001
possibility of professional promotion	90 (36.7%)	21 (56.8%)	69 (33.2%)	0.006
contact with patients	177 (71.4%)	16 (42.1%)	161 (76.7%)	<0.001
position responsibility	86 (34.8%)	12 (31.6%)	74 (35.4%)	0.649
patient safety	133 (54.3%)	34 (89.5%)	99 (47.8%)	<0.001
appreciation by other medical employees of hospitals	135 (54.4%)	22 (57.9%)	113 (53.8%)	0.642

*n* (%): Number of respondents with percentages in each category, i.e., “yes” and “no”. For “yes”, answers “strong impact” and “very strong impact” and for “no”, answers “hard to say”, “no impact”, “definitely no impact” were combined together.

**Table 5:** The odds of willingness to work as an infection control and prevention nurse in relation to the importance of the listed issues.

Reason for the Justification of Willingness to Work as an ICPN	Univariate Model		Multivariate Model	
	OR (95%CI)	*p*-Value	OR (95%CI)	*p*-Value
salary	4.2 (2–8.7)	<0.001	3.6 (1.5–8.8)	0.005
possibility of professional development	3.8 (1.6–9)	0.002	3.6 (1.3–9.9)	0.013
possibility of professional promotion	2.6 (51–5.4)	0.007		
contact with patients	0.2 (0.1–0.5)	<0.001	0.1 (0.1–0.4)	<0.001
position responsibility	0.8 (0.4–1.8)	0.649		
patient safety	9.3 (3.2–27.1)	<0.001	8.9 (2.9–27.8)	<0.001
appreciation by other medical employees of hospitals	1.2 (0.6–2.4)	0.642		

OR: Odds ratio; CI: Confidence interval.

## Appendix A

**Table A1:** Reasons for interest in working as an infection prevention and control nurse.

Factors Determining the Willingness to Work as an Infection Control Nurse	Number	Significant Impact	Some Impact	Hard to Say	No Impact	Definitely No Impact
	*n*	%				
working as an IC nurse gives the opportunity to cooperate and interact with various professional groups in the hospital	38	50.0	39.5	7.9	2.6	-
IC nurse work greatly improves patient safety	38	60.5	28.9	10.6	-	-
working as an IC nurse gives you the opportunity to develop professionally	38	31.6	50.0	15.8	-	2.6
the work of the IC nurse involves a wide range of qualifications and the ability to make decisions independently	37	35.2	45.9	18.9	-	-
the IC nurse position is situated high in the organizational hierarchy of hospitals, which is associated with prestige	38	44.7	34.2	21.1	-	-
working as an IC nurse does not involve the shift work system, making it easier to reconcile it with private obligations	38	34.2	42.1	18.4	5.3	-
In your opinion, working as an IC nurse is associated with a greater variety of tasks and professional responsibilities	38	26.3	34.2	39.5	-	-
the work of an IC nurse is highly regarded by other medical staff in hospitals	38	31.6	26.3	31.5	5.3	5.3
work as an IC nurse gives you the opportunity to get promoted	37	40.5	16.2	40.6	-	2.7
this nursing job is associated with a higher salary than in other nursing jobs	37	29.7	21.6	29.7	16.3	2.7
work as an IC nurse is associated with less contact with patients	38	21.1	21.1	15.8	26.2	15.8
working as an IC nurse is associated with less responsibility than in other nursing jobs	38	15.8	15.8	15.8	26.3	26.3
obtaining a specialization and working as an IC nurse is easier than other nursing specialties	38	10.5	21.1	28.9	23.7	15.8

**Table A2:** Reasons for the lack of interest of nursing students in employment as an infection prevention and control nurse.

Reasons for the Lack of Interest of Nursing Students in Employment as an Infection Control Nurse	Number	Significant Impact	Some Impact	Hard to Say	No Impact	Definitely No Impact
	*n*	%				
work as an IC nurse does not allow contact with patients/provides limited possibilities of contact with patients	210	40.5	36.2	10	7.6	5.7
work as an IC nurse is associated with a greater burden of office/administrative work	211	34.6	41.3	10.4	8.5	5.2
work as an IC nurse is not appreciated by other employees of medical hospitals	210	22.9	31.0	28.5	12.4	5.2
working as an IC nurse does not enable the desired professional development	210	23.3	30.5	24.8	16.2	5.2
work as an IC nurse has little impact on real/daily work in wards	207	16.6	35.7	23.6	16.9	7.2
work as an IC nurse is perceived as a burdensome obstacle to everyday work by most medical staff	208	22.1	28.8	29.4	12.5	7.2
work as an IC nurse has little effect on improving patient safety	207	17.9	30.0	27.5	14.0	10.6
work as an IC nurse is associated with lower pay than other nursing positions	209	5.7	14.4	35.9	25.4	18.7
work as an IC nurse does not offer career opportunities	208	6.3	26.9	30.7	23.1	13.0
work as an IC nurse is associated with greater responsibility than at other nursing positions	209	14.8	20.6	31.1	19.1	14.4
